# Microfluidic Device Type Improves Heart mRNA Delivery *In Vivo*

**DOI:** 10.1021/acs.langmuir.5c02612

**Published:** 2025-10-02

**Authors:** Elisa Schrader Echeverri, Hyejin Kim, Bora Jang, Avraham Shakked, Christian Park, Kyung In Baek, Leandro Choi, Dong Won Kang, Ruei-Chun Hung, Kiyoung Jeong, Hannah E. Peck, Ananda R. Podilapu, Karen E. Tiegreen, Philip J. Santangelo, Hanjoong Jo, James E. Dahlman

**Affiliations:** Wallace H. Coulter Department of Biomedical Engineering, Emory University School of Medicine and Georgia Institute of Technology, Atlanta, Georgia 30322, United States

## Abstract

To improve lipid nanoparticle (LNP)-mediated delivery to nonliver tissues, scientists modify LNP chemistry or add targeting ligands. One underexplored alternative is to change the formulation process that creates the LNP. Here, we report that an LNP formulated with a herringbone mixer led to 2-fold more heart delivery than the same LNP formulated with a bifurcating mixer. The two LNPs had similar biophysical traits, yet they adsorbed different protein coronas, suggesting that the effect was driven by systemic physiology. By using spatial transcriptomics to track LNP delivery in a mouse model of atherosclerosis, we found that the improved LNP delivered mRNA to diseased regions and cardiomyocytes after an intravenous injection. These data suggest that it is possible to increase heart delivery *via* nanoparticle processing.

## Graphical abstract

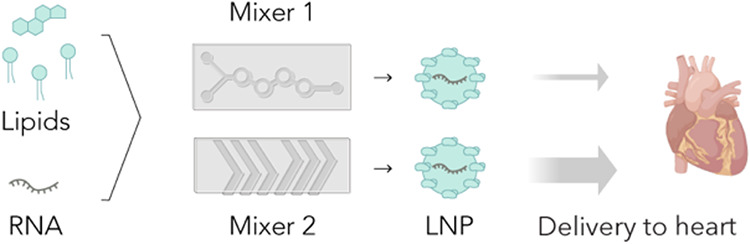

## Introduction

Lipid nanoparticles (LNPs) deliver therapeutic RNA in three Food and Drug Administration (FDA)-approved drugs.^1–3^ These therapies hint at the potential impact of next-generation RNA drugs, including those that can target nonliver organs after an intravenous administration.^4^ To improve nonliver delivery, scientists often add targeting ligands such as antibodies^5–7^ or change one of the components that constitute the LNP: the ionizable lipid, the cholesterol, the poly­(ethylene-glycol) (PEG)-lipid, or the helper lipid. Modifying the individual components or their molar ratios has been shown to alter tropism.^8,9^ For example, laboratories have reported that using a positively charged helper lipid shifted LNP tropism to the lungs,^10–14^ and scientists demonstrated that including both a positively charged cholesterol and a positively charged helper lipid led to the discovery of an LNP termed LNP^++^, which had heart tropism.^15^

One possibility for changing LNP tropism is to alter the process used to create the LNP. LNPs are typically formulated using microfluidic mixers,^16–22^ which are often either chaotic herringbone mixers or bifurcating mixers. We reasoned that switching to a different formulation device could affect *in vivo* tropism, even if the LNPs created by the device had similar biophysical traits. To test this hypothesis, we formulated LNP^++^ using both mixers and characterized the resulting LNP size, polydispersity, encapsulation efficiency, zeta potential, protein corona, and subsequent *in vivo* tropism after intravenous injection. We found that the LNPs had similar biophysical traits but dissimilar protein coronas and heart tropism. Subsequent studies in a mouse model of atherosclerosis revealed that the herringbone-formulated LNP delivered mRNA to plaques and cardiomyocytes after an intravenous injection. Taken together, these data support the hypothesis that formulation processes can independently alter the *in vivo* tropism of seemingly similar LNPs.

## Experimental Section

### Nanoparticle Formulation

Lipid nanoparticles were formulated in two different microfluidic devices by mixing a citrate buffer solution containing cre mRNA (provided by Dr. Philip Santangelo at Emory University) with a solution containing cKK-E12 (Cayman Chemicals), DC-cholesterol HCl, C_14_PEG_2000_, and DOTAP (all other lipids purchased from Avanti Polar Lipids) at a molar ratio of 30.5:27:2.5:40 in 100% ethanol. The formulation was performed at a 10:1 total lipid:RNA mass ratio and a flow rate ratio of 3:1 citrate phase:lipid phase. In the herringbone mixer, the ethanol solution flowed at 200 μL/min and the citrate solution at 600 μL/min; in the NxGen device, the flow rates were 3000 and 9000 μL/min, respectively. One microfluidic device contained a herringbone mixing structure as previously reported;^19^ the other was the Ignite chip from Precision Nanosystems with a bifurcating-based NxGen mixing design. After formulation, the LNPs were dialyzed in a 20 kDa microdialysis plate (Thermo Scientific) and sterilized with a 0.22 μm filter.

### Nanoparticle Characterization

The polydispersity and hydrodynamic diameter of the particles were measured using dynamic light scattering (DLS), and their zeta potential was measured using electrophoretic light scattering (ELS) on a Malvern Zetasizer Advanced-Pro (red). Eight hundred microliters of the particles were loaded into a Folded Capillary Cell (DTS1070). The settings used were the following: an absorbance of 0.01, a material refractive index of 1.4, a dispersant viscosity of 0.882 cP, a refractive index of 1.22, and a dielectric constant of 79.

### Encapsulation Efficiency

To determine the concentration of LNPs, we used the Quant-it RiboGreen RNA Assay kit (Thermo Fisher R11490). The assay was run in duplicates. Fifty microliters of LNPs at 6 ng/μL were added to 50 μL of either 1× TE buffer (Thermo Fisher) or 2% Triton X-100 (Sigma-Aldrich). After 10 min of incubation at 37 °C, 100 μL of RiboGreen agent diluted at 1:100 was added to each well, and fluorescence was quantified using a plate reader (BioTex Synergy H4 Hybrid). The excitation wavelength was 485 nm, and the emission wavelength was 528 nm.

### mRNA Synthesis

Cre mRNA was synthesized as described in previous work.^23^ First, the mRNA sequence was human codon optimized using the Integrated DNA Technologies (IDT) website. It was then ordered as a DNA gBlock to contain a 5′ UTR with the Kozak sequence, a 3′ UTR derived from the mouse α-globin sequence, and extensions to allow for Gibson assembly. It was then cloned to make a PCR-amplified pMA7 vector through Gibson assembly using NEB Builder with a 2 molar excess of insert. The Gibson assembly reaction transcripts were 0.8% agarose gel-purified prior to the reactions. The resulting plasmids from each colony were Sanger sequenced to confirm their sequence identity. Notl-HF (New England Biolabs, NEB) was used to linearize the plasmids overnight at 37 °C and then purified by ammonium acetate (Thermo Fisher Scientific) precipitation before being rehydrated with nuclease-free water. After this, *in vitro* transcription (IVT) was done overnight at 37 °C with the HiScribe T7 kit (NEB) following the manufacturer’s instructions (N1-methyl-pseudouridine modified). To then remove the template, the RNA was treated with DNase I (Aldevron) for 30 min and then purified by lithium chloride precipitation (Thermo Fisher Scientific). Next, it was heat-denatured for 10 min at 65 °C before being modified with a Cap1 structure using guanylyl transferase (Aldevron) and 2′-*O*-methyltransferase (Aldevron), and polyadenylated enzymatically (Aldevron). The mRNA was then purified as before, treated with alkaline phosphatase (NEB), and purified again. A NanoDrop was used to measure the concentration of the mRNA. The stocks were 3–5 mg/mL. Gel electrophoresis was used to ensure the purity of the RNA.

### TNS Assay

The p*K*_a_ of herringbone-LNP^++^ and NxGen-LNP^++^ was measured as described in previous work.^24^ In summary, a solution made of 10 mM sodium phosphate, 10 mM sodium borate, 10 mM sodium citrate, and 150 mM sodium chloride was made and pH adjusted using sodium hydroxide and hydrogen chloride to create solutions with pH ranging between 3 and 12. The assay was run in triplicate for each LNP. Ninety microliters of the pH-adjusted buffer was added to a 96-well plate. Then, 2 μL of 300 mM 2-(p-toluidino)-6-naphthalene sulfonic acid and 3.26 μL of LNP were added to each well. The plate was incubated for 5 min on a shaker. The fluorescence absorbance was then quantified using an excitation wavelength of 325 nm and an emission wavelength of 435 nm (BioTek Synergy H4 Hybrids). The data were used to create a sigmoidal plot of fluorescence *versus* buffer pH. The logarithm of the inflection point of the curve was taken as the p*K*_a_ of the LNP.

### Characterization of the Hard Protein Corona

The method used to quantify the enrichment of the plasma proteins on the surface of the LNPs was adapted from the method used before.^25,26^ To summarize it here, the assay was run in quadruplets, and mouse plasma (*n* = 5) was combined with LNP samples at a 1:1 volume ratio. The samples were mixed and left to incubate for 15 min at 37 °C. The mixture was then loaded onto a cushion made of 0.7 M sucrose in Milli-Q water. It was then centrifuged for 1 h at 4 °C at 15,300*g*, then washed with 1× PBS and centrifuged again at the same speed for 5 min. It was washed a total of three times. RIPA buffer was used to resuspend the pellet, and a Pierce BCA Protein Assay kit was used to quantify the protein concentration. Next, the Bolt LDS Sample Buffer (Novex) and 1% dithiothreitol was used to dilute the samples. They were then boiled for 5 min at 90 °C and left at room temperature for 5 min. The samples were combined with iodoacetamide and mixed at room temperature for 30 min. Thirty-two microliters of the samples was loaded onto a Bolt 10% Bis-Tris Plus WedgeWell Gel and they were run into the gel at 200 V. SimplyBlue Safe Stain was used to stain the gels for 1 h to be able to visualize the proteins. The gels were then destained for 1 h, and the bands were isolated with a sterile razor and put into tubes with 10 μL of Milli-Q water. The samples were stored at 4 °C until they were submitted to the Emory University Proteomics Core for mass spectrometry analysis.

### In-Gel Digestion

Gel bands were minced and destained twice with 1:1 (v/v) 50 mM ammonium bicarbonate (ABC) and acetonitrile (CAN). Finally, the gels were washed with 100% CAN and dried for 30 min in a SpeedVac. Ten nanograms per microliter of trypsin solution (diluted in ABC) was added to the dried gel and digested overnight. The next day, 50% CAN with 5% formic acid was used twice for peptide extraction followed by a final step with 100% CAN. The peptide solution was dried under vacuum, and a cleanup step was done with stage tip. The resulting peptides were dried under a vacuum.

### LC-MS/MS

Dried peptides were resuspended in loading buffer (0.1% trifluoroacetic acid, TFA) and were separated on a self-packed 25 cm column (100 μm internal diameter packed with 1.7 μm Waters CSH beads) using an Easy-nLC 1200 or Dionex 3000 RSLCnano liquid chromatography system. The liquid chromatography gradient started at 1% buffer B (80% acetonitrile with 0.1% formic acid) and ramped to 5% in 6 s. This was followed by a 35 min linear gradient to 23% B, 6 min to 35% B, and finally a 4 min 99% B flush. Orbitrap Lumos Tribrid mass spectrometer was used to acquire all mass spectra. The spectrometer was operated in data-dependent mode in top speed mode with a cycle time of 3 s. Survey scans were collected in the Orbitrap with a 60,000 resolution, 400 to 1600 *m*/*z* range, 400,000 automatic gain control (AGC), 118 ms max injection time, and rf lens at 30%. Higher energy collision dissociation (HCD) tandem mass spectra were collected in the Orbitrap with a collision energy of 30%, an isolation width of 1.6 *m*/*z*, an AGC target of 50,000, and a max injection time of 54 ms. Dynamic exclusion was set to 40 s with a 10 ppm mass tolerance window.

### MaxQuant

Spectra were searched using the search engine Andromeda, integrated into MaxQuant, against Uniprot/Swissprot 2020 Mouse database (17,041 target sequences). Methionine oxidation (+15.9949 Da), asparagine and glutamine deamidation (+0.9840 Da), and protein N-terminal acetylation (+42.0106 Da) were variable modifications (up to 5 allowed per peptide); cysteine was assigned as a fixed carbamidomethyl modification (+57.0215 Da). Only fully tryptic peptides were considered with up to 2 missed cleavages in the database search. A precursor mass tolerance of ±20 ppm was applied prior to mass accuracy calibration and ±4.5 ppm after internal MaxQuant calibration. Other search settings included a maximum peptide mass of 6000 Da, a minimum peptide length of 6 residues, 0.05 Da tolerance for Orbitrap, and 0.6 Da tolerance for ion trap MS/MS scans. The false discovery rates for peptide spectral matches, proteins, and site decoy fraction were all set to 1%. Quantification settings were as follows: requantify with a second peak finding attempt after protein identification has completed; match MS1 peaks between runs; a 0.7 min retention time match window was used after an alignment function was found with a 20 min RT search space. Quantitation of proteins was performed using summed peptide intensities given by MaxQuant. The quantitation method considered only razor plus unique peptides for protein level quantitation.

### Nanoparticle Administration into Mice

LNPs were administered intravenously *via* the tail vein of the mice at 1.0 mg RNA/kg. The syringes used were the Monoject 28G × 1/2″ insulin syringes (Covidien 8881600004).

### Animal Experiments

All animal experiments were performed in compliance with the Emory University Institutional Animal Care and Use Committee (IACUC). Ai14 mice, Cre reporter mice,^27^ were bred at the Emory University animal facility. In all experiments, *N* = 3–5 mice per group aged 5–8 weeks were injected intravenously through the lateral tail vein, unless noted otherwise.

### Organ Isolation and Staining for Flow Cytometry and FACS

Mice were sacrificed 72 h after injection. They were perfused with 10 mL of 1× PBS in the right atrium. The lung, liver, and heart were isolated immediately after perfusion. The lung and liver were finely cut and placed into a digestive enzyme mixture composed of Collagenase Type I, Collagenase XI, and Hyaluronidase (all purchased from Sigma-Aldrich). The heart was minced finely and placed into a digestive enzyme mixture composed of Collagenase Type I, Collagenase XI, Hyaluronidase, and Collagenase Type IV. All three tissues were digested for 30 min at 37 °C and 550 rpm. They were then passed through 70 μm cell strainers (Biologix Research Company, 15–1070), and the strainers were then washed with 7 mL 1× PBS. They were centrifuged for 5 min at 500*g*. We removed the supernatant and added 250 μL of 1% TruStain FcX (anti-mouse CD16/32) Antibody (BioLegend 101319). After letting them sit at 4 °C in the TruStain FcX Antibody for 15 min, cells were stained to identify specific cell types for flow cytometry on the BD FACSymphony in the Emory + Pediatrics/Winship Flow cytometry core. The antibodies used were CD31 for endothelial cells (clone 390, BioLegend), CD45.2 for immune cells (clone 104, BioLegend), TER119 for red blood cells (Stemcell Technologies), and LIVE/DEAD Fixable Aqua Dead Cell stain (Invitrogen). Flow cytometry gating strategies are shown in Figures S1–S3.

### Organ Isolation for *In Situ* Spatial Transcriptomics Analysis Platform, Xenium

Mice were sacrificed 72 h after LNP administration and perfused with ice-cold PBS followed by 4% paraformaldehyde and then drop-fixed overnight. The hearts were then sectioned and placed on Xenium slides (10X Genomics) and processed according to the manufacturer’s protocol. The slides were then stained for tdTomato (Anti-RFP pAb, PM005, MBL Life Science) and DAPI according to the IF protocol from 10X Genomics. The Xenium Explorer software package was used to analyze and cluster the single-cell data for each section, according to the gene expression available in Table S1.

### Atherosclerosis Disease Model in Mice

Hypercholesterolemia was induced by injecting AAV8-PCSK9 (1 × 10^11^ VG/mouse, Vector Biolabs #AAV8-D377Y-mPCSK9) *via* tail vein and subsequently feeding with a high-fat Western diet as previously described.^28^ Partial carotid ligation (PCL) surgery to induce disturbed flow (d-flow) in the left common carotid artery (LCA) was carried out 1 week after AAV8-PCSK9 injection.^29–33^

### Nanoparticle Experiment in Atherosclerotic Mice for Histology

Two weeks post-PCL LNPs were administered intravenously *via* the tail vein of the mice at 1.0 mg/kg with PBS as a control as previously stated. The syringes used were Monoject 28G × 1/2″ insulin syringes. Mice were euthanized 3 days after injection for immunofluorescence analyses. To determine EC-targeted Cre mRNA delivery and consequent tdTomato expression, two segments of the LCA and a section of the contralateral RCA were longitudinally sectioned, the aortic arch was collected *en face* and visualized using the well-established Goat anti-tdTomato antibody (AB8181–200, OriGene) and Donkey anti-Goat Alexa Fluor 594 (A-11058, Thermo Fisher Scientific).^32^ To further recapitulate EC-targeted delivery, tdTomato expression in endocardium, aortic valve endothelial cells, aortic wall, and sinus was sectioned and imaged in cross-sectional views. Blocking of nonspecific epitopes and antibody incubations were performed by using PBS supplemented with 0.1% Triton X-100 + 5% BSA as previously described. The stained sections were DAPI-mounted (F6057, Sigma-Aldrich) for imaging. Stained sections were imaged by an inverted wide-field microscope (Keyence #BZ-X800, Japan) as previously described. Following the image acquisition, blind deconvolution and dehazing of out-of-focus illumination, background removal, image superimposition, and automated image stitching were performed using custom-written MATLAB algorithms (MathWorks, MA), ImageJ, and Fiji (NIH, MD). The average fluorescence intensity in each field of view was analyzed by using ImageJ and Fiji to quantify changes in tdTomato expression in CAs. tdTomato induction in the aortic arch for each group of mice was quantified using Otsu’s method.^32,34,35^ Intensity histogram of tdTomato^+^ binary masks in the aortic arch was normalized against segmented autofluorescence from the elastic lamina for quantification.

## Results and Discussion

### Comparing Effects of Different Formulation Methods on LNP Physical Characteristics

We first formulated LNP^++^ using a herringbone-based polydimethylsiloxane (PDMS) microfluidic device^19^ (Figure S4) or a ring-based Precision Nanosystems NxGen microfluidic device.^36^ We focused on LNP^++^ since it was reported to have tropism to the heart, lung, and liver^15^; this presented an opportunity to measure tropism across multiple tissues. We diluted the ionizable lipid cKK-E12,^37^ cholesterol, C_14_PEG_2000_, and 1,2-dioleoyl-3-trimethylammonium-propane (DOTAP) in 100% ethanol and separately diluted chemically modified mRNA encoding Cre recombinase in 10 mM pH 3 citrate buffer. In the herringbone mixer, the ethanol solution flowed at 200 μL/min and the citrate solution at 600 μL/min; in the NxGen device, the flow rates were 3000 and 9000 μL/min, respectively. We then compared the resulting hydrodynamic diameter, polydispersity index, zeta potential, p*K*_a_, and encapsulation efficiency (Figure 1a). Both formulations of LNP^++^ had a hydrodynamic diameter near 60 nm, a polydispersity index near 0.2, a p*K*_a_ near 7.0, and a zeta potential near 0 mV. We therefore concluded there were no overt changes to LNP biophysical traits as a function of the formulation device.

**Figure 1:**
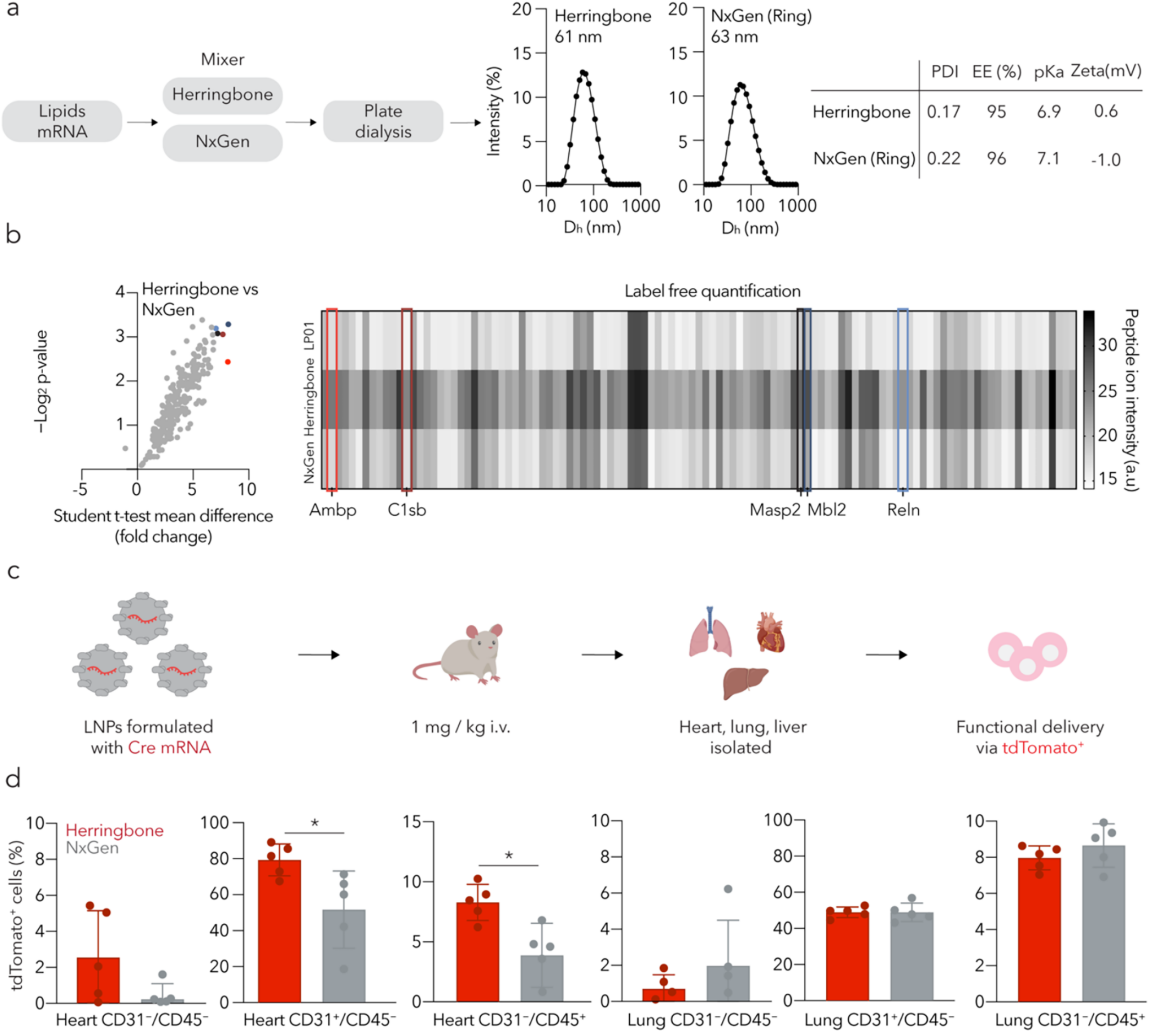
Varying the LNP formulation method does not change nanoparticle biophysical traits but leads to differential *in vivo* tropism. (a) LNP^++^ was formulated with a herringbone mixer or a NxGen bifurcating mixer, and each formulation biophysically compared. Two LNP^++^ formulations were shown to be almost physically identical. PDI, polydispersity index; EE, encapsulation efficiency; Zeta, zeta potential. (b) A second round of characterization was performed to compare protein corona composition. When comparing peptide ion intensity (a.u.), herringbone-LNP^++^ had a higher enrichment in all proteins compared to NxGen-LNP^++^, and a difference was seen in label-free quantification based on peptide ion intensity between all three LNPs. Analyzed by Student’s *t*-test. (c) Both LNP^++^ carrying Cre mRNA were then injected intravenously at a 1 mg/kg dose into separate groups of Ai14 mice. 72 h after injection, the heart, lung, and liver were isolated and processed for flow cytometry. (d) tdTomato expression was quantified in different cell types within the heart and lung, and was greater in heart for herringbone-LNP^++^. Data are presented as mean ± SD (*N* = 5). * *p* < 0.05, analyzed by unpaired *t*-test.

### Comparing Effects of Different Formulation Methods on Protein Corona and *In Vivo* Delivery

We then analyzed the molecules that adsorbed onto the LNPs after they were exposed to plasma, since the protein corona affects LNP behavior *in vivo*.^26,38^ After mixing the LNPs with mouse plasma and using liquid chromatography–mass spectrometry and MaxQuant as a quantification method, we analyzed the bound proteins (Figures 1b and S5). Despite the biophysical similarity of the LNPs, we found that those formulated with the herringbone mixer (herringbone-LNP^++^) were enriched in all identified proteins, relative to the LNPs formulated with the NxGen mixer (NxGen-LNP^++^). Of these 256 proteins, we focused on five that were enriched by more than 7-fold: reelin, complement C1s-C subcomponent, mannan-binding lectin serine protease, mannose-binding protein C, and α-1-microglobulin. When we analyzed these proteins, we noted that three were previously identified in adult mouse hearts, while the other two have been reported in the liver.^39^ We then used label-free quantification based on the peptide ion intensity and observed that 145 proteins had a significantly higher intensity in herringbone-LNP^++^ than in NxGen-LNP^++^ and none had a lower intensity. Taken together, these protein corona data led us to hypothesize that herringbone-LNP^++^ might exhibit different tropism.

To test this hypothesis, we formulated both LNP^++^ with mRNA encoding Cre recombinase and intravenously injected Ai14 mice^27^ at a 1.0 mg/kg dose. In these reporter mice, cells express tdTomato if Cre mRNA is translated into functional Cre protein, which excises a stop codon preceding the tdTomato construct. After 72 h, we sacrificed the mice and isolated the heart, lung, and liver, then prepared the tissues for flow cytometry (Figure 1c). Consistent with the protein corona data, the percentage of tdTomato^+^ cells in the heart was higher in mice treated with herringbone-LNP^++^ (Figure 1d). We observed statistically significant increases in the percentage of tdTomato^+^ cells in all three heart cell types. One potential explanation for this was that herringbone-LNP^++^ was more potent globally. We therefore analyzed delivery in three lung cell types and three liver cell types (Figures 1d and S6). Of these six cell types, only one—liver CD31^+^CD45^–^ cells—had statistically significant increased delivery with herringbone-LNP^++^. These data did not support the hypothesis that herringbone-LNP^++^ had a higher global potency.

When analyzing the flow cytometry data in mice treated with herringbone-LNP^++^, we noted low but measurable delivery to CD31^–^CD45^–^ cell types. This combination of markers could include many heart cell types, including cardiomyocytes and cardiac fibroblasts, which have been traditionally difficult to transfect yet are important targets for genetic medicines. Given that further identifying CD31^–^CD45^–^ cells from the heart using traditional flow cytometry is difficult,^40^ we developed a single-cell spatial transcriptomics-based approach to measure LNP delivery.^41,42^ We formulated LNP^++^ to carry Cre mRNA with the herringbone device, injected it intravenously into Ai14 mice at 1.0 mg/kg dose, waited for 72 h, and then fixed the hearts with paraformaldehyde. After sectioning the hearts, we identified individual cell types using spatial transcriptomics on a Xenium system, then overlaid tdTomato immunofluorescence data onto these cells. This allowed us to quantify delivery in cell types that are difficult to isolate using flow cytometry. We observed a high percentage of tdTomato^+^ endothelial cells and a lower percentage of immune cells (Figures 2 and S7–S8). Since blood flow varies in different regions of the heart, we used spatial transcriptomics to evaluate delivery in the different regions. Interestingly, for both endothelial cells and immune cells, we observed more transfection in the ventricles than in the atria.

**Figure 2:**
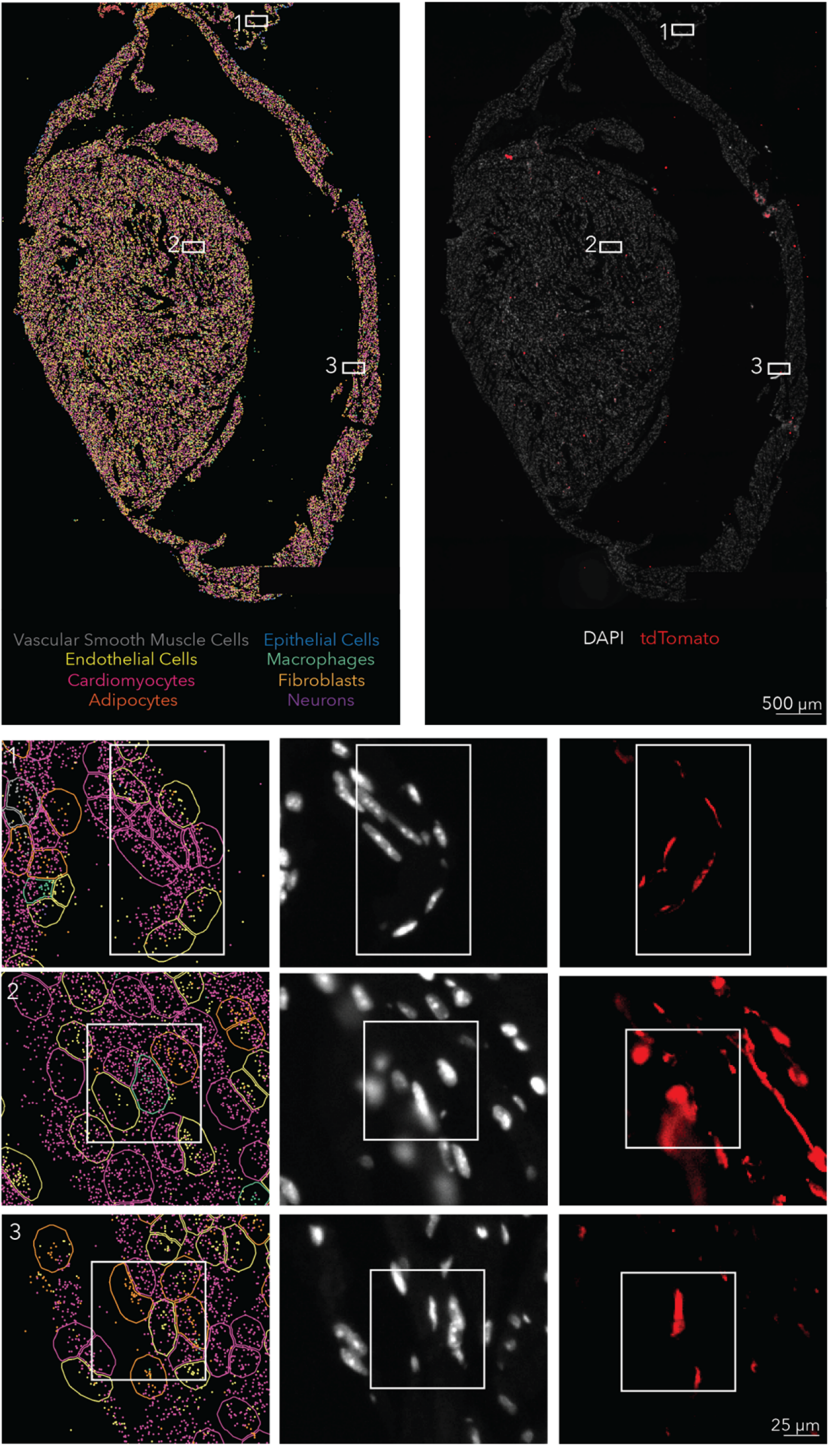
Single-cell spatial quantification of herringbone-LNP^++^. The single-cell spatial imaging system Xenium was used to analyze the delivery of herringbone-LNP^++^ at better resolution. The results confirmed the flow cytometry results and showed most of the delivery to occur in the ventricle.

### Functional Delivery of LNP-Mediated Cre mRNA into Atherosclerotic Mice

The fact that regions of the heart associated with different blood flow showed different LNP delivery led us to propose that nanoparticles could also deliver mRNA in a mouse model of atherosclerosis, which is characterized in part by disturbed flow.^33^ Since atherosclerotic lesions develop heterogeneously, making cells from the lesions hard to differentiate from cells in healthy heart tissues using flow cytometry, we again used spatial transcriptomics to evaluate delivery. We first generated the atherosclerosis-like phenotype by administering an AAV8 encoding PCSK9 under the liver-specific promoter apolipoprotein enhancer/alpha-1 antitrypsin to Ai14 mice that were also given a high-fat diet.^28^ These hypercholesterolemic mice then underwent partial carotid artery ligation surgery to induce disturbed flow, thereby accelerating the development of atherosclerotic lesions.

We then used the herringbone mixer to formulate LNP^++^ so that it carried Cre mRNA and intravenously injected it at a dose of 1.0 mg/kg into the mice. We observed a high percentage of tdTomato^+^ cells (Figures 3a and S9). We found high percentages of tdTomato^+^ cells in both the lower and the greater curvature of the aortic arch. We compared delivery in the left carotid artery and the right carotid artery, since the model requires a partial ligation of the left artery to accelerate disease (Figures 3b and S10). We did not observe any difference, suggesting that endothelial cell delivery is possible across different regions of the heart. We then used spatial transcriptomics to quantify delivery in cardiomyocytes and fibroblasts (Figure S11), two clinically relevant cell types that were likely within the CD31^–^CD45^–^ cell population. The cardiomyocytes were identified using previously identified genes Mylk3, Myoz2, Pgam2, and Ttn;^43–45^ fibroblasts were identified using Htra3, Lum, Mfap4, Mfap5, and Fibin.^46–49^ We found tdTomato^+^ cardiomyocytes in the ventricles and tdTomato^+^ fibroblasts in both the ventricles and the left atrium. We also analyzed the liver as a known off-target organ (Figures 3c and S12). Consistent with the flow data in healthy mice, we observed delivery to hepatic endothelial cells as well as central, midlobular, and portal hepatocytes.

**Figure 3:**
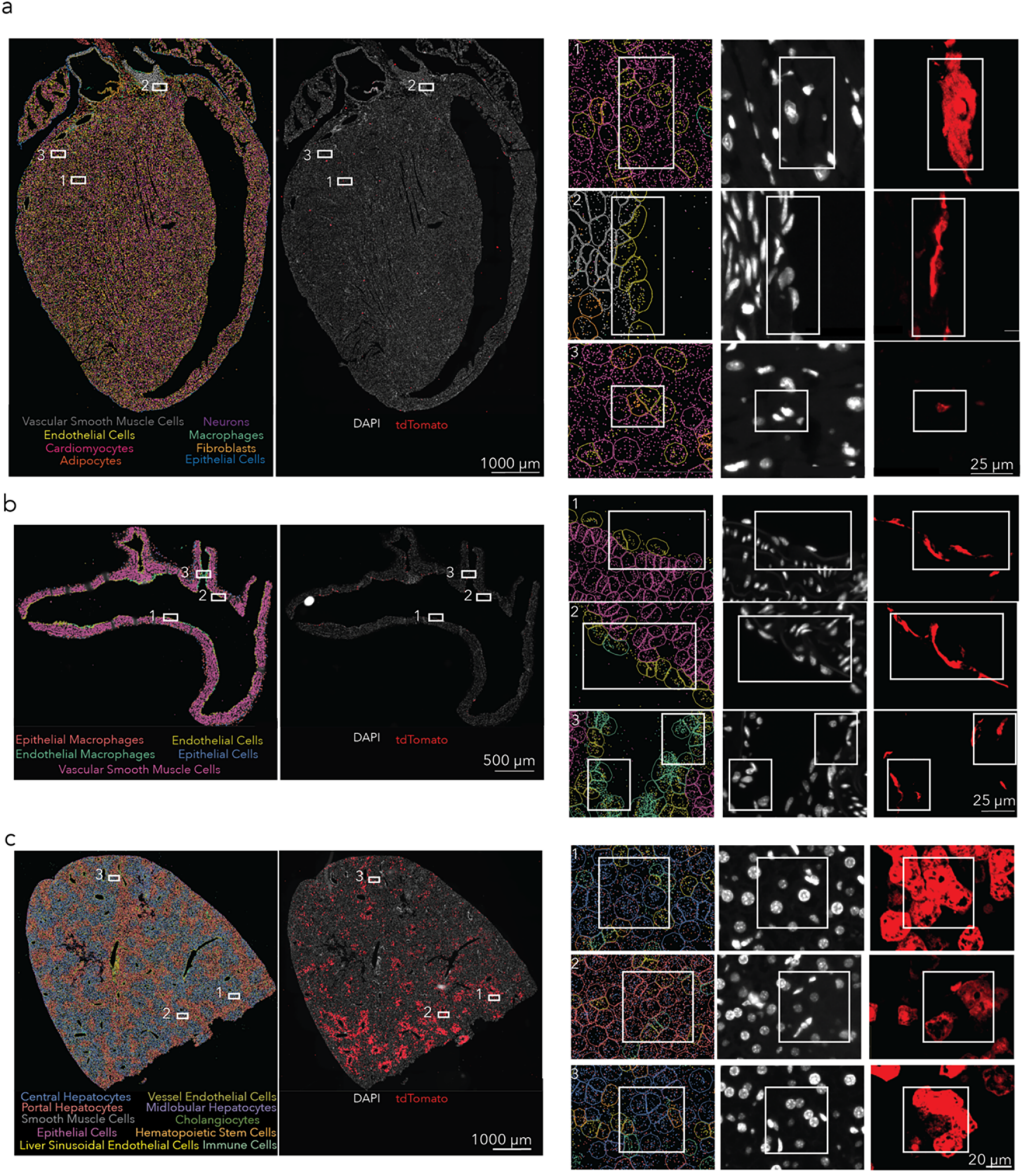
Herringbone-LNP^++^-treated atherosclerotic heart and aortic arch analysis with single-cell spatial imaging. (a) tdTomato expression in the heart was detected in cardiomyocytes, endothelial cells, and fibroblasts. (b) tdTomato expression in the aortic arch was detected in both lower and greater curvature as well as in the carotid arteries. The two cell types expressing tdTomato were endothelial cells and macrophages on the endothelial side of the vasculature. (c) tdTomato expression was detected in the various types of hepatocytes as well as in the vessel endothelial cells.

Finally, to confirm delivery using a complementary approach, we again administered the herringbone-LNP^++^ to Ai14 mouse models of atherosclerosis and then analyzed heart delivery using immunofluorescence. Three days after administering a 1.0 mg/kg dose of Cre mRNA, we found substantial tdTomato expression in the heart (Figure 4). Taken together, these data led us to conclude that herringbone-LNP^++^ delivered mRNA to the heart in the presence of atherosclerosis.

**Figure 4:**
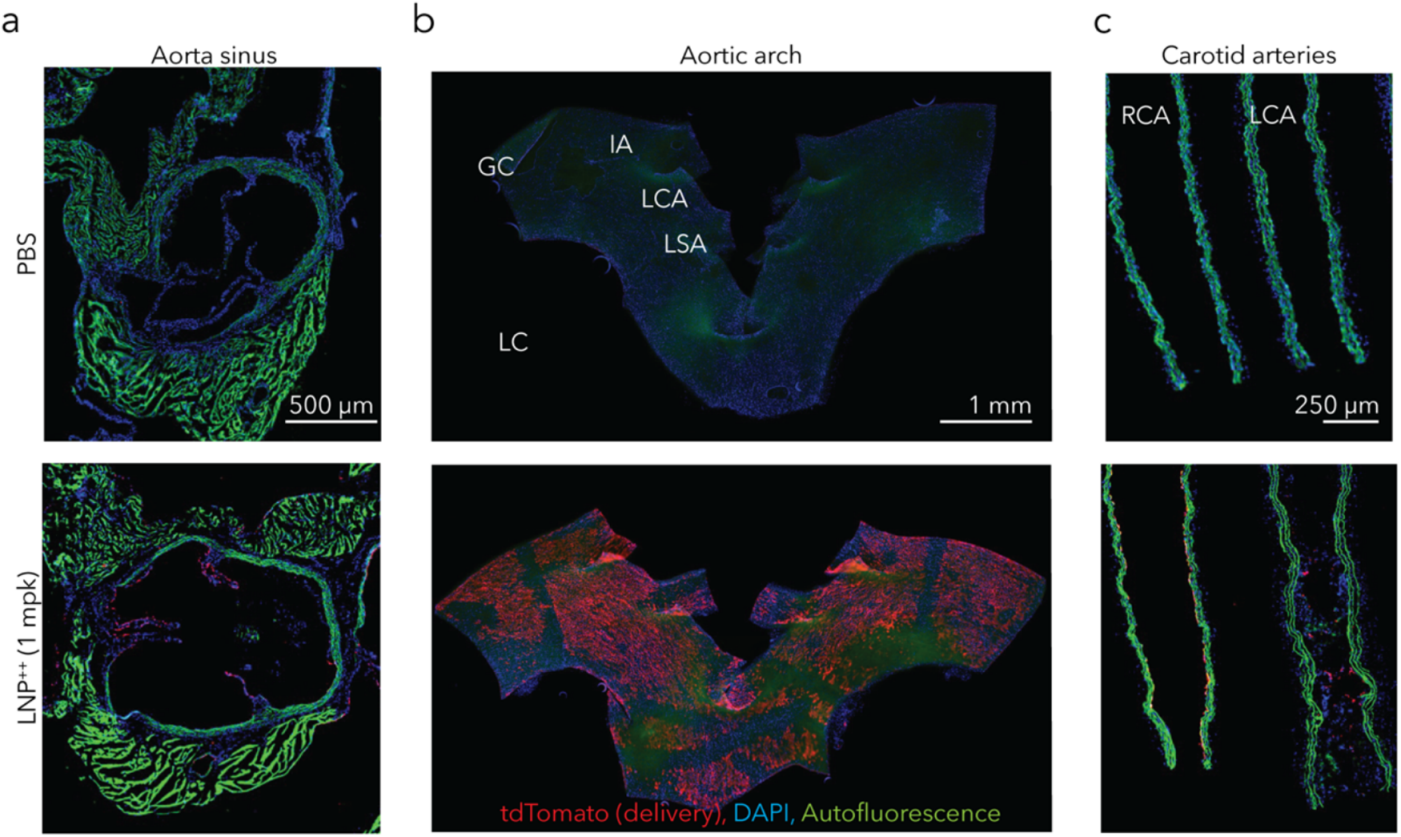
Herringbone-LNP^++^ delivery to an atherosclerotic mouse model *via* tdTomato expression. Mice administered herringbone-LNP^++^ carrying Cre mRNA at 1 mg/kg dose. (a) Aortic sinus shows tdTomato expression on the endothelial level. mpk, mg/kg. (b) The *en face* view of the aortic arch shows tdTomato expression uniformly across the arch. GC, greater curvature; LC, lesser curvature; IA, innominate artery; LCA, left carotid artery; LSA, left subclavian artery. (c) The right carotid artery (RCA) and left carotid artery (LCA) show little difference between the two.

## Conclusions

Scientists change the LNP activity by adding targeting ligands or altering the LNP chemical composition. By contrast, seemingly subtle differences in the LNP formulation process have not been considered to be a similarly important variable. Here, we present evidence that *in vivo* LNP delivery can be affected by the microfluidic mixer type used to formulate the particles. Interestingly, these differences in tropism were not driven by overt changes in the biophysical parameters. Instead, we found evidence that the protein corona adsorbed onto the particle differed. We observed protein corona components that have been studied for their role in tropism,^50^ including apolipoproteins (ApoC3, ApoB-100, ApoM) and complement factors (C1s-b, Cfd, C4b, C1r-a, C1r-b, C1s-a), which were enriched in herringbone-LNP^++^ compared to NxGen-LNP^++^. These data are consistent with lines of evidence that protein corona can substantially influence LNP behavior,^21^ and suggest that the changes in protein corona profiles can be used as an early readout for understanding whether a given formulation parameter should be tested *in vivo*. They also suggest two ideas that could be important for nanoparticle design. The first is that different formulation parameters should be tested not only for their influence on LNP biophysical traits but also for their effect on delivery to on- and off-target organs *in vivo.* The second is that scaling up the production of an LNP from mouse to nonhuman primate or human doses may be complicated by small formulation variables. This second point may be especially important given that not all microfluidic mixers are commercially available in large scale; scientists may therefore prefer completing early studies with microfluidic devices that can be scaled up in order to prevent later-stage surprises.

Previous work on how mixing types and microfluidic mixing parameters affected LNP performance in mice provided a strong foundation by characterizing *in vivo* delivery using bioluminescence, a tissue-level delivery readout.^21^ Here we complemented that work by reporting delivery at the single-cell level, finding that spatial transcriptomics may be useful for identifying delivery in cell types that are hard to isolate with flow cytometry. Specifically, although flow cytometry did enable us to identify delivery in CD31^–^CD45^–^ cells, spatial transcriptomics identified that delivery occurred in cardiomyocytes and fibroblasts. Similarly, the spatial readouts allowed us to evaluate whether delivery changed in regions of disturbed flow (specifically, plaques) and in different chambers within the heart. We anticipate that future studies will utilize the techniques we developed to quantify delivery alongside the spatial data sets to test hypotheses in other hard-to-digest tissues.

It is important to acknowledge the limitations of this work. First, while the expression of tdTomato gives us a good idea of whether a particular LNP can functionally deliver nucleic acids to cells, it does not test the ability to produce a therapeutic effect. It will be important to identify mRNA-based targets that could slow down or reverse atherosclerosis and then test them in these disease models. In this study, we focused on evaluating LNP^++^ formulated using a herringbone microfluidic device in an atherosclerotic model, as it demonstrated higher potency in healthy mice. One limitation of this approach is that LNPs may behave differently under diseased conditions; therefore, further investigation will be necessary to assess their efficacy more comprehensively in pathological settings. Additionally, a limitation of using PBS as a control is that it does not account for potential effects of the mRNA cargo itself; thus, future studies using a scrambled or control mRNA will be necessary to more comprehensively evaluate its function. Another limitation is that the behavior of an LNP can differ across species^51^ and it is unclear how well delivery in mice predicts delivery in nonhuman primates or pigs, which are considered good models for humans. Despite these limitations, we believe that these data convey the importance of understanding the subtle differences between microfluidic mixers and the need to characterize how they affect protein corona and *in vivo* delivery early in the LNP development process.

## Supplementary Material



## Data Availability

All data needed to evaluate the conclusions in the paper are present in the main text and Supporting Information or available from the authors.

## Notes

Flow cytometry gating strategies; herringbone mixer design; raw data of proteomics data; flow cytometry results for liver tissue; tdTomato quantification in heart mice administered with herringbone-LNP^++^; Xenium single-cell images of tissue administered with herringbone-LNP^++^; quantification of tdTomato-expressing cells from Xenium analysis; PBS-treated liver tissue analyzed on Xenium; transcript groups used to identify cell types in Xenium-analyzed tissues (PDF)
