# Supplementary material for: Microfluidic Device Type Improves Heart mRNA Delivery In Vivo
Source: Langmuir. 2025 Oct 2;41(40):27123–32. doi: 10.1021/acs.langmuir.5c02612 (PMC12529952; doi:10.1021/acs.langmuir.5c02612)
Supplement: Supplementary file 1 [file la5c02612_si_001.pdf]

Supporting Information for

Microfluidic device type improves heart mRNA delivery *in vivo*

Elisa Schrader Echeverri[1], Hyejin Kim[1], Bora Jang[1], Avraham Shakked[1], Christian Park[1], Kyung In Baek[1], Leandro Choi[1], Dong Won Kang[1], Ruei-chun Hung[1], Kiyoung Jeong, Hannah Peck[1], Ananda R. Podilapu[1], Karen E. Tiegreen[1], Philip J. Santangelo[1], Hanjoong Jo[1], and James E. Dahlman[1]

[1]Wallace H. Coulter Department of Biomedical Engineering, Emory University School of Medicine and Georgia Institute of Technology, Atlanta, GA, 30322

Correspondence: james.dahlman@emory.edu

Table of Contents

Figure S1: Flow cytometry gating of LNP-mRNA delivery in the heart. 2

Figure S2: Flow cytometry gating of LNP-mRNA delivery in the liver 3

Figure S3: Flow cytometry gating of LNP-mRNA delivery in the lung 4

Figure S4: Design and dimensions of the herringbone mixer 5

Figure S5: Raw data (peptide ion intensity) of proteomics gene data 6

Figure S6: Flow cytometry liver delivery comparison of herringbone-LNP^{++} and NxGen-LNP^{++} 7

Figure S7: tdTomato quantification in heart from mouse administered with herringbone-LNP^{++} .8

Figure S8: PBS-treated heart analysis with Xenium system 9

Figure S9: PBS-treated heart from atherosclerotic mouse 10

Figure S10: PBS-treated aortic arch from atherosclerotic mouse 11

Figure S11: Quantification of tdTomato-expressing cells from Xenium images and analysis 12

Figure S12: PBS-treated liver from atherosclerotic mouse 13

Table S1: Transcript groups used to identify cell types in the tissues analyzed with the Xenium system. 14

Reference 15

Supplementary Figures

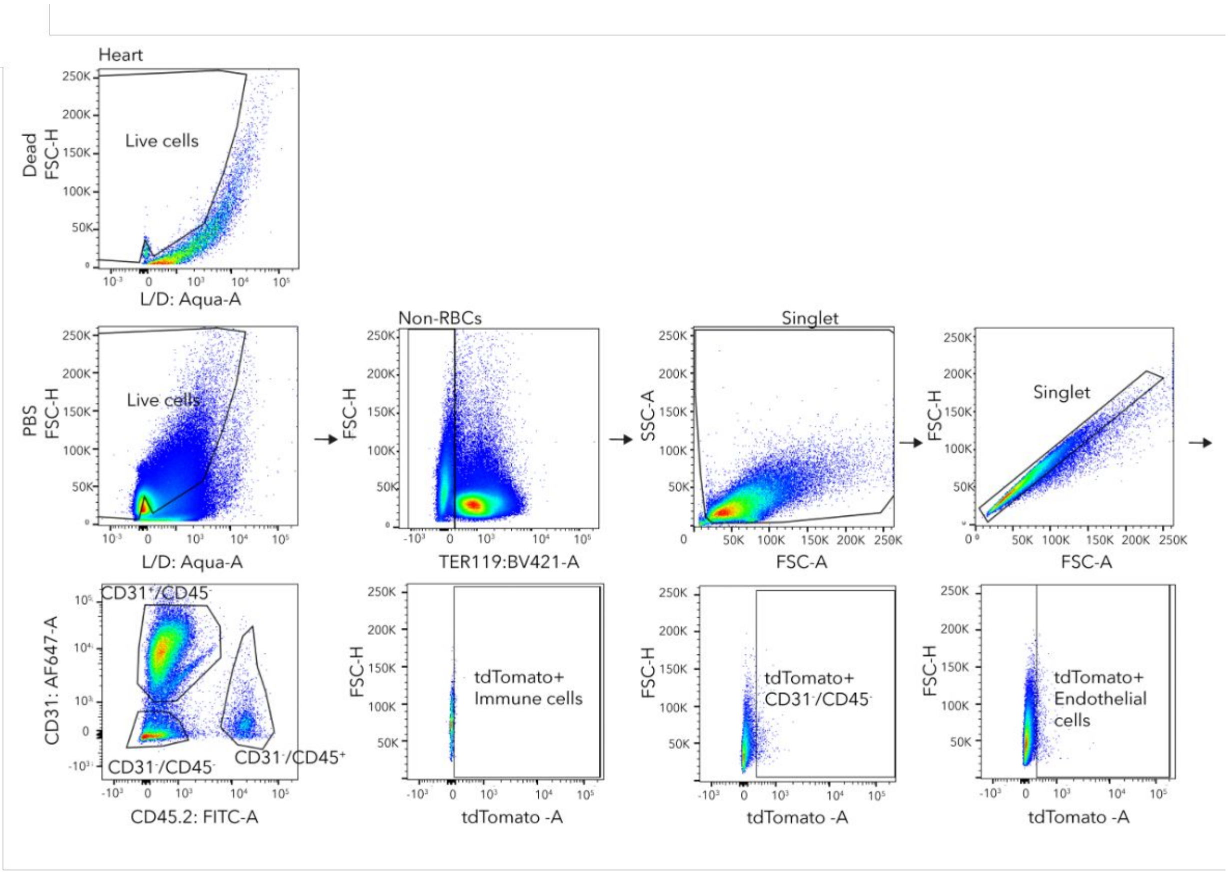

Figure S1: Flow cytometry gating of LNP-mRNA delivery in the heart.

Representative gating strategy for heart tissue. RBC: red blood cells; L/D: live/dead.

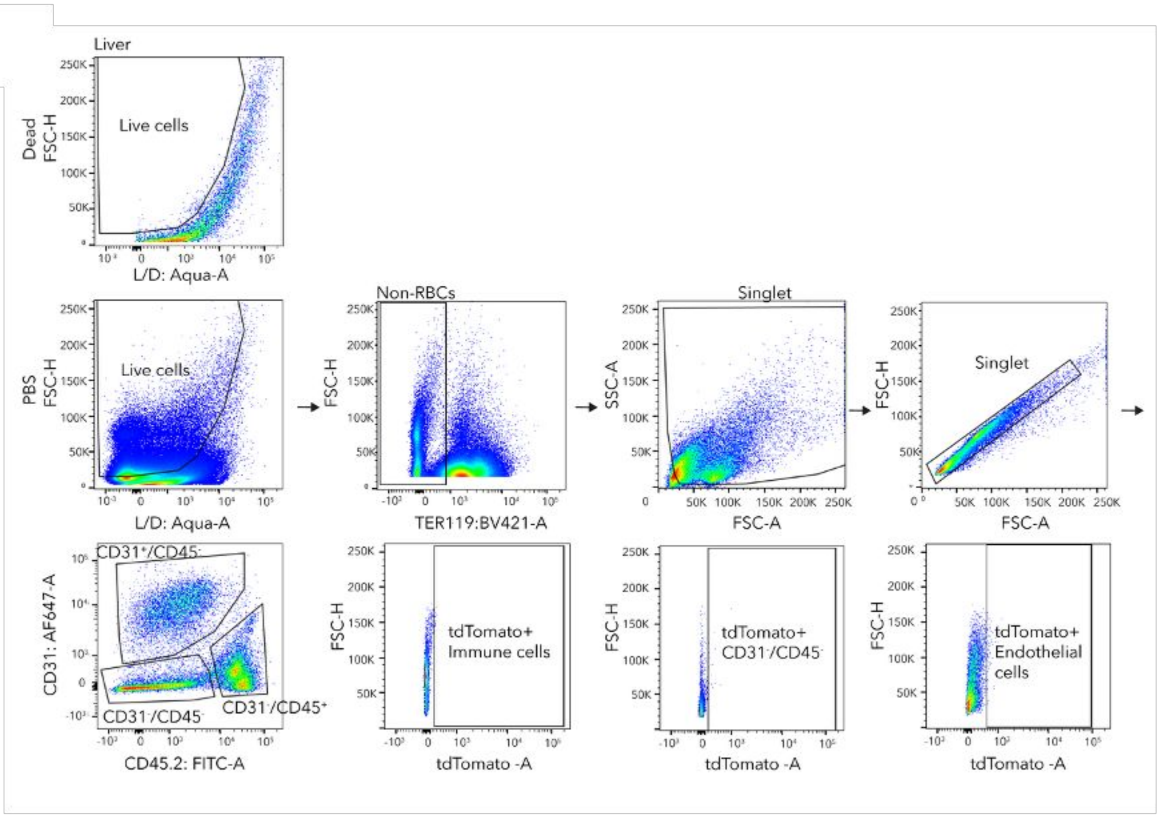

Figure S2: Flow cytometry gating of LNP-mRNA delivery in the liver

Representative gating strategy for liver tissue. RBC: red blood cells; L/D: live/dead.

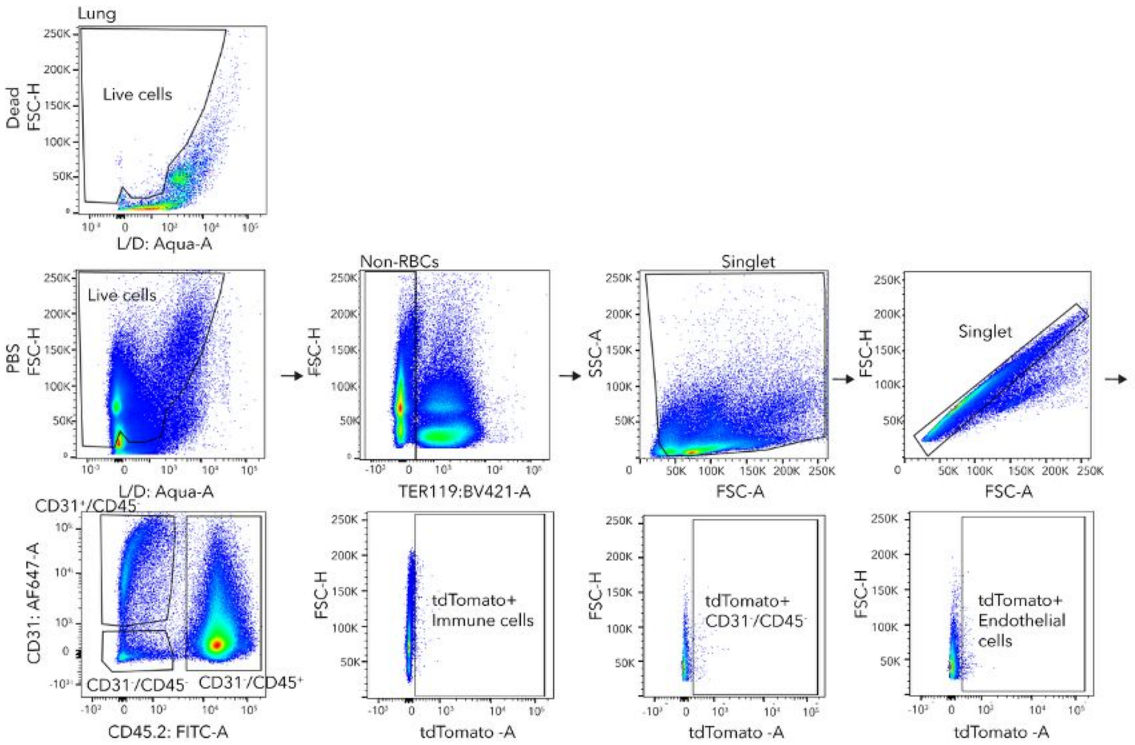

Figure S3: Flow cytometry gating of LNP-mRNA delivery in the lung

Representative gating strategy for lung tissue. RBC: red blood cells; L/D: live/dead.

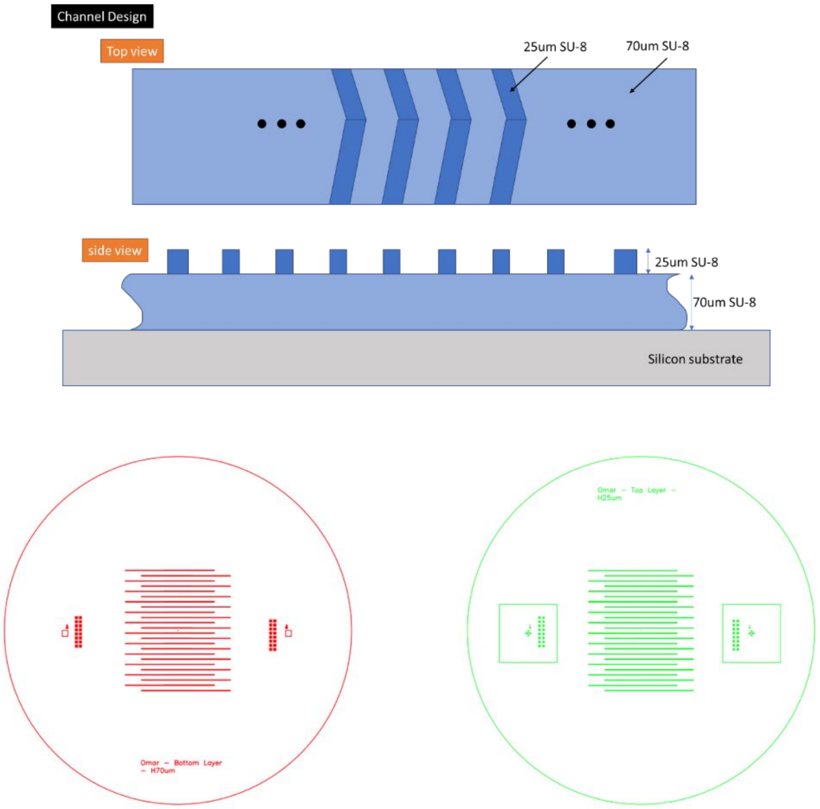

Figure S4: Design and dimensions of the herringbone mixer

Periodic trenches and ridges were asymmetrically arranged on the floor of the channel to promote chaotic mixing of the solutions as previously reported[1].

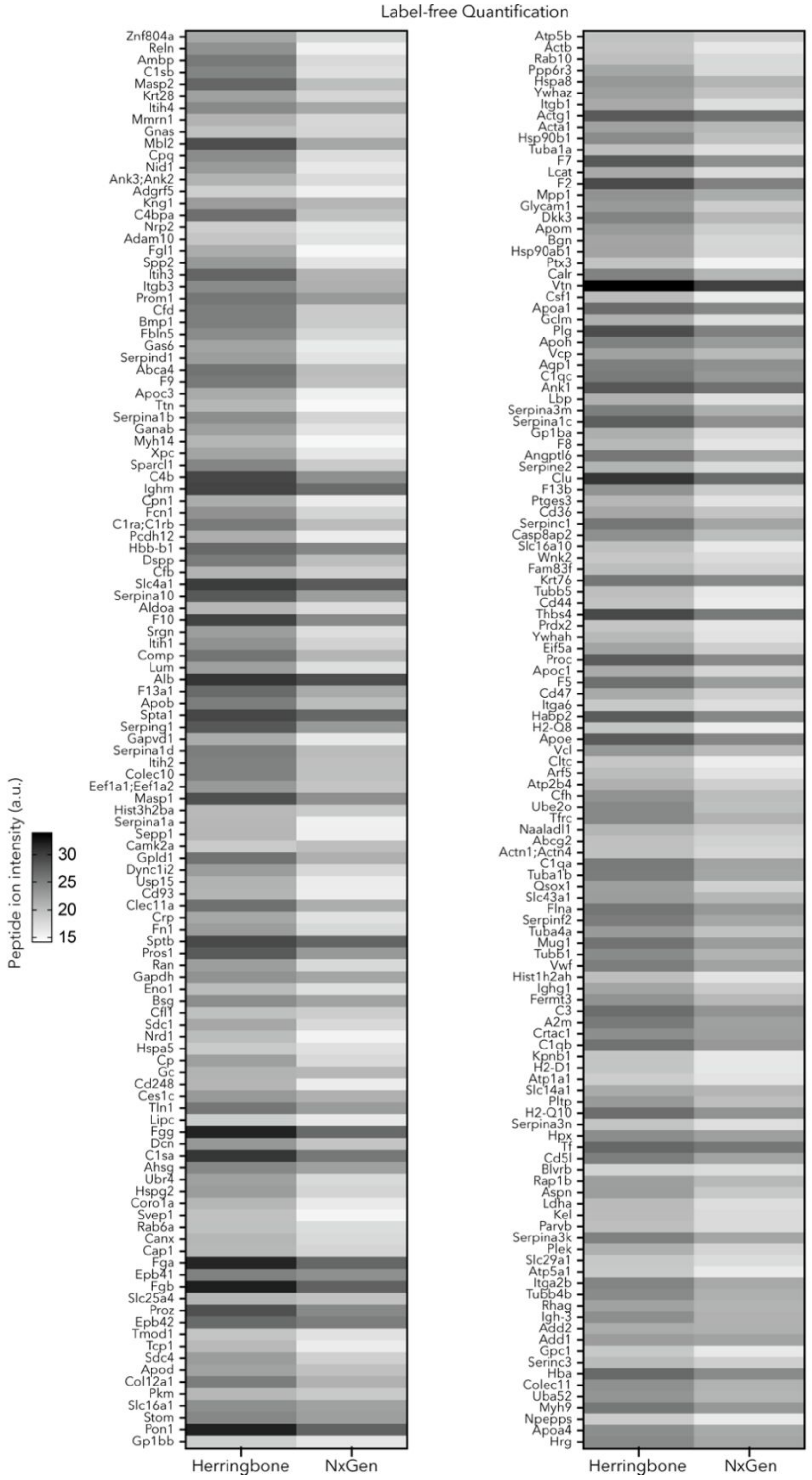

Figure S5: Raw data (peptide ion intensity) of proteomics gene data

Raw data (peptide ion intensity) of proteomics gene data of herringbone-LNP^{++} and NxGen-LNP^{++}. With an n=4 the various amounts of each gene are represented.

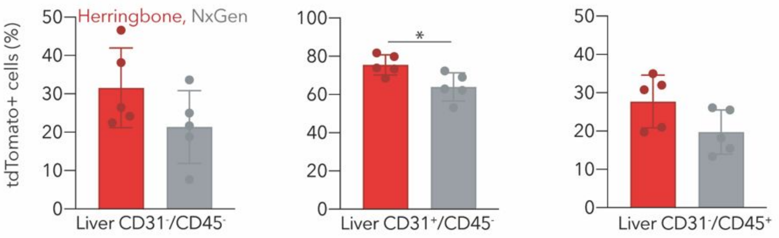

Figure S6: Flow cytometry liver delivery comparison of herringbone-LNP^{++} and NxGen-LNP^{++}

Flow cytometry tdTomato quantification in the liver. Ai14 mice were intravenously injected with the herringbone-LNP^{++} and NxGen-LNP^{++} containing Cre mRNA. The tdTomato expression in different cell types was quantified and compared using flow cytometry. The only cell type that showed a statistically significant difference was the CD31^{+}/CD45-. Data are presented as mean ± SD (N=5). * $p < 0.05$, analyzed by unpaired t-test.

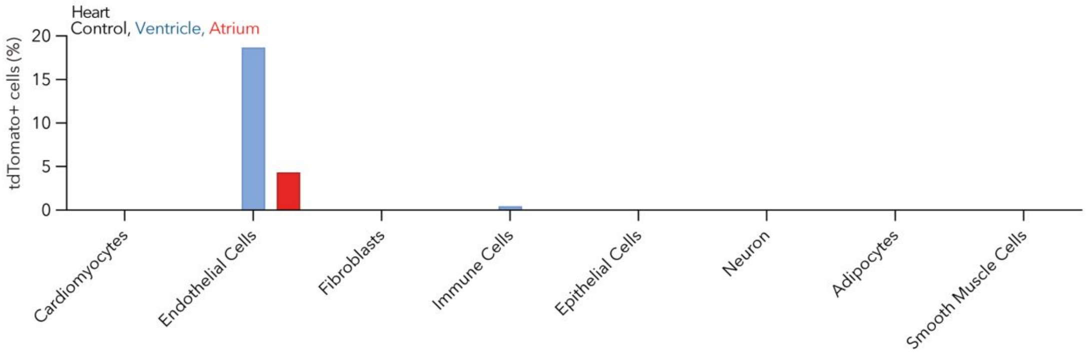

Figure S7: tdTomato quantification in heart from mouse administered with herringbone-LNP^{++}

Control was a tissue slice from an untreated mouse, which showed no tdTomato expression.

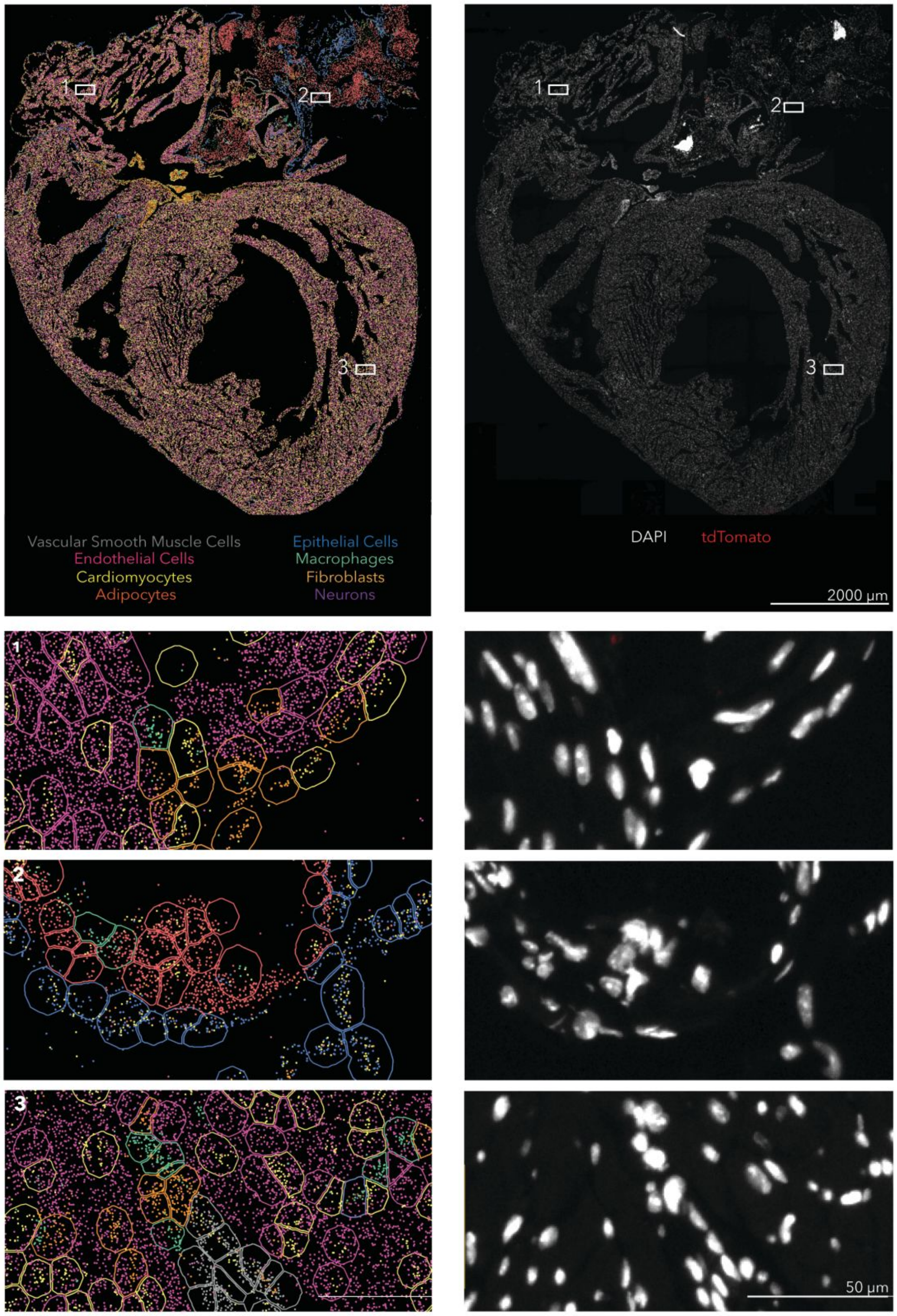

Figure S8: PBS-treated heart analysis with Xenium system

No tdTomato expression was detected, and all the same cell subtypes were seen as in the LNP-treated mouse.

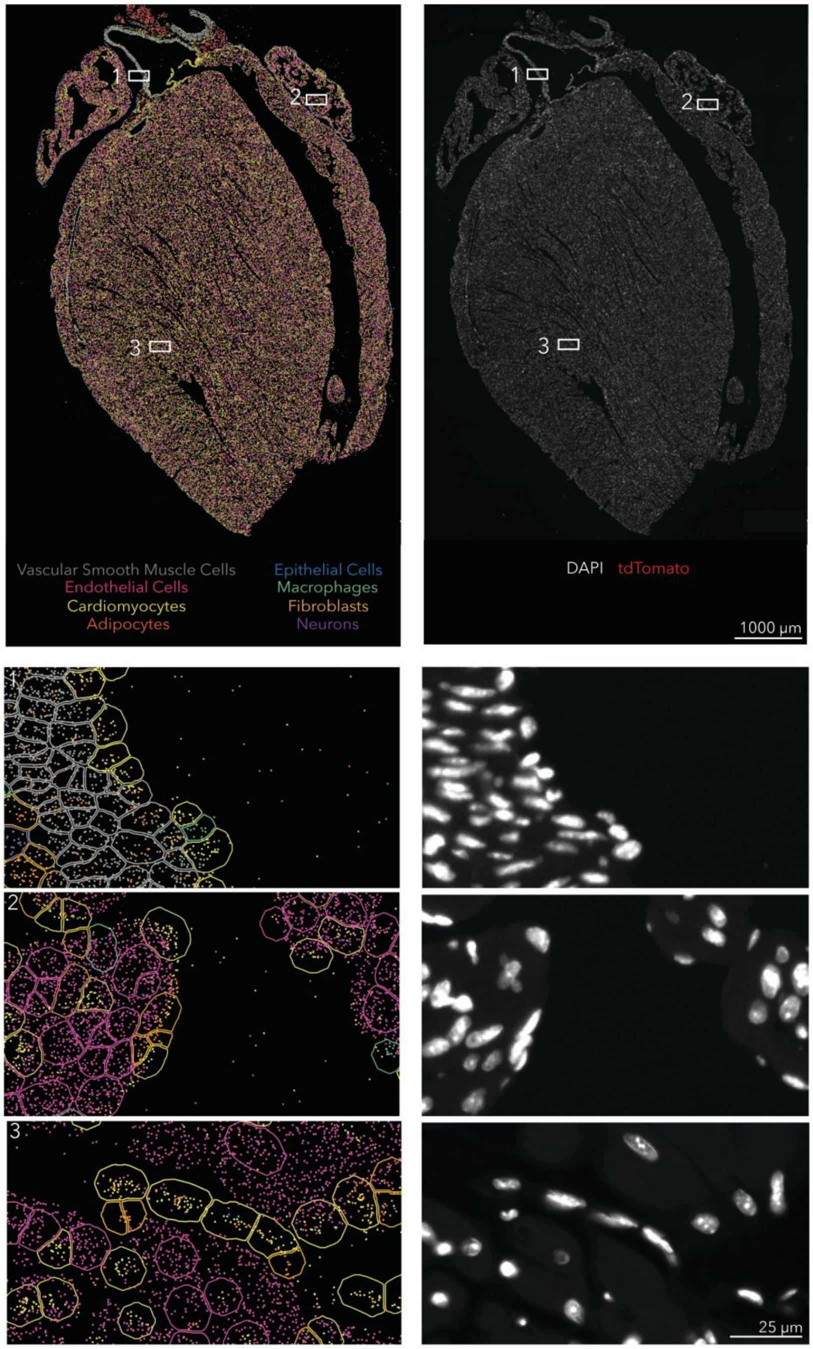

Figure S9: PBS-treated heart from atherosclerotic mouse

No tdTomato expression was detected, and all the same cell subtypes were seen as in the LNP-treated sample.

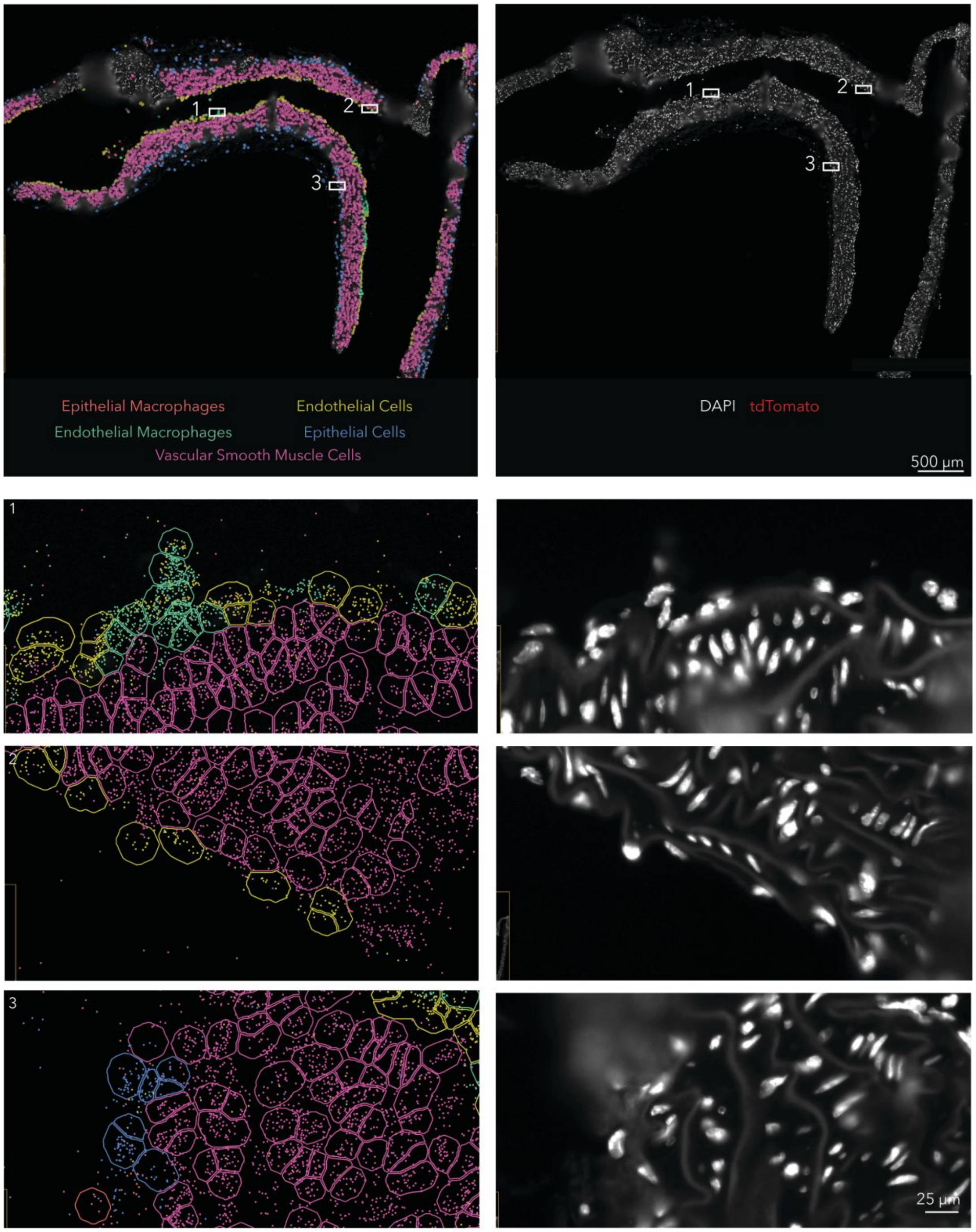

Figure S10: PBS-treated aortic arch from atherosclerotic mouse

No tdTomato expression was detected, and all the same cell subtypes were seen as in the LNP-treated sample.

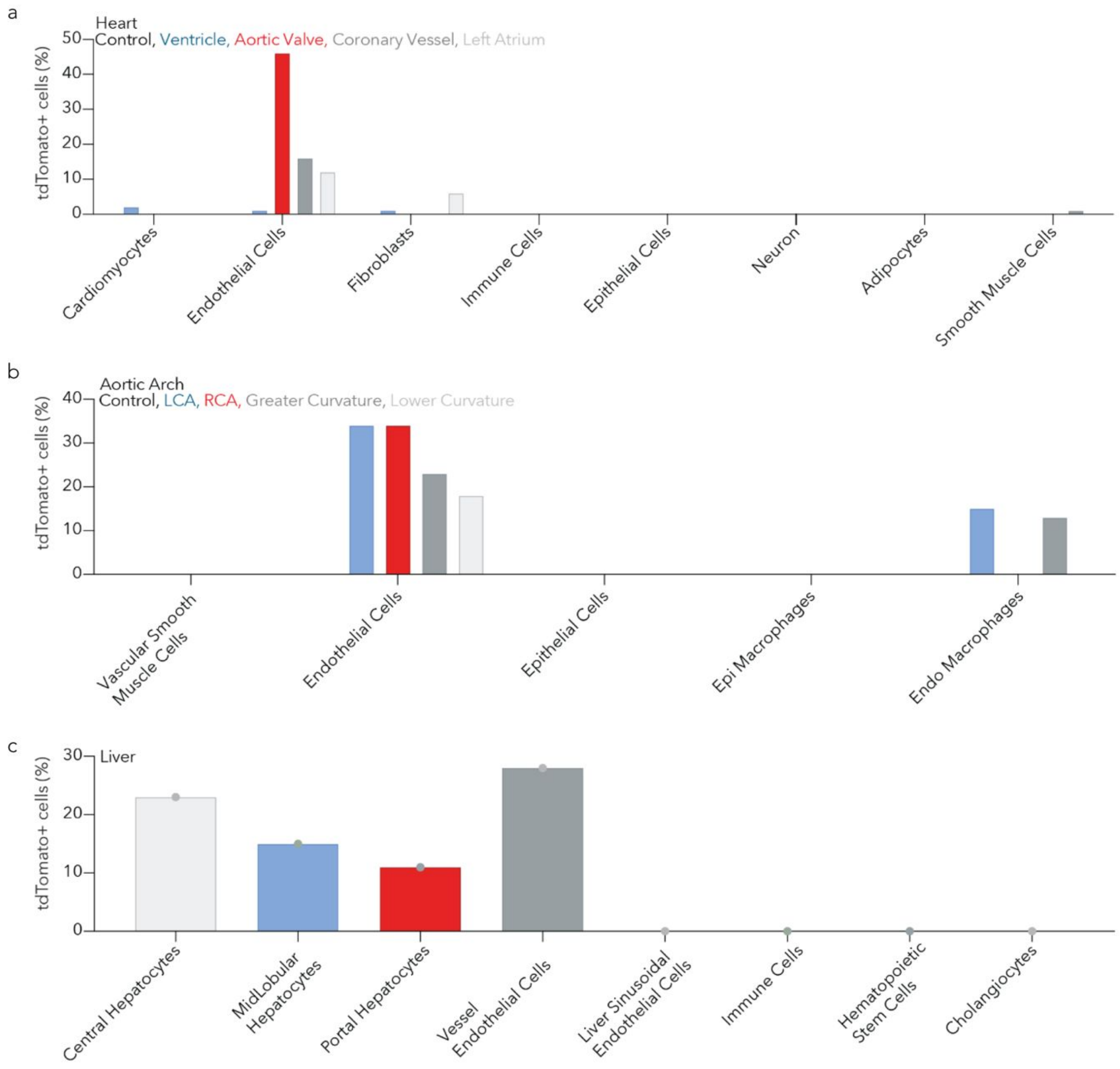

Figure S11: Quantification of tdTomato-expressing cells from Xenium images and analysis

Controls were tissue slices of (A) heart, (B) aortic arch, and (C) liver that came from PBS-treated mice. Those tissues showed no tdTomato expression.

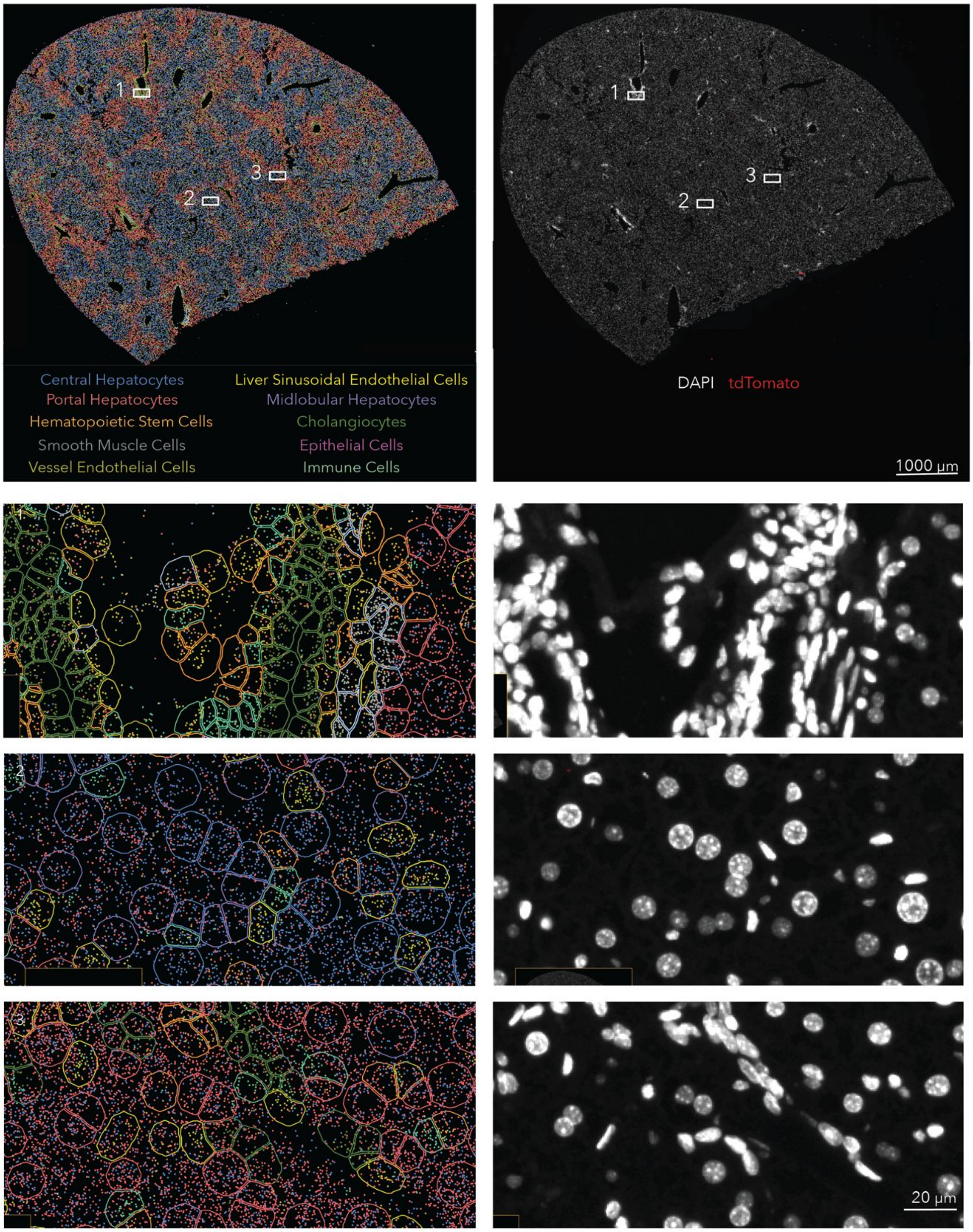

Figure S12: PBS-treated liver from atherosclerotic mouse

No tdTomato expression was detected, and all the same cell subtypes were seen as in the LNP-treated sample.

Table S1: Transcript groups used to identify cell types in the tissues analyzed with the Xenium system.

Graph-based clustering was used in addition to these transcript groups to identify different cell types. **(A)** heart, **(B)** aortic arch, and **(C)** liver.

a Heart

Cell Type	Transcript ID
Adipocytes	Car3
	Hp
	Ppp1r1a
	Retn
	Slc27a2
Cardiomyocytes	Mylk3
	Myoz2
	Pgam2
	Ttn
Atrial Cardiomyocytes	Slc17a7
Endothelial Cells	Vwf
	Plvap
	Gpihbp1
	Kdr
	Aqp1
	Podxl
Epithelial Cells	Krt19
	Krt8
	Upk1b
	Upk3b
Fibroblasts	Htra3
	Lum
	Mfap4
	Mfap5
	Fibin
Macrophages	F13a1
	Ms4a6c
	Ms4a6b
	Folr2
	Mpeg1
	Ms4a7
Sensory Neurites	Kcna1
	Chl1
	Plp1
	Ptn
Neurons	Celf3
	Gap43
	Snap25
T-cells	Cd3d
	Cd8a
Vascular Smooth Muscle Cells	Myh11
	Tagln

b Aortic Arch

Cell Type	Transcript ID
Vascular Smooth Muscle Cells	Myh11
	Tagln
	Cnn1
Endothelial Cells	Vwf
	Tie1
	Podxl
	Ctla2a
	Sfrp1
	Eng
	Cavin2
Epithelial Cells	Serpinf1
	Lum
	F3
	Pi16
	c3
	dpt
Endothelial Macrophages	Atp6v0d2
	Mpeg1
	Cd5l
Epithelial Macrophages	F13a1
	Pf4
	Lyve1

c Liver

Cell Type	Transcript ID
Portal Hepatocytes	Apoc2
	Pck1
	Sdc1
	Hpx
Central Hepatocytes	Car3
	Hsd3b4
	Slc27a2
Liver Sinusoidal Endothelial Cells	Kdr
	Lyve1
	Aqp1
Vessel Endothelial Cells	Vwf
Hematopoietic Stem Cells	Fbln5
	Mamdc2
	Lhx2
	Colec11
	Lum
	Mfap4
Immune Cells	Ms4a6b
	Ms4a6c
	Ms4a7
	Ctss
	s100a4
	Epsti1
Smooth Muscle Cells	Myh11
	Myl9
	Tagln
Cholangiocytes	Epcam
	Muc1
	Tspan8
	Slc5a1
Epithelial Cells	Upk3b
	Igfbp6
	Bst1
	Upk1b
	Nbl1
Neutrophils	Csf3r
	Cxcr2
	Mmp9

Reference

1. Chen, D. et al. Rapid discovery of potent siRNA-containing lipid nanoparticles enabled by controlled microfluidic formulation. *J Am Chem Soc* **134**, 6948-6951 (2012).
